# Central Granular Cell Odontogenic Tumor: Case Report with Literature Review of Cases Reported in the Last 71 years

**DOI:** 10.30476/dentjods.2022.94300.1774

**Published:** 2023-03

**Authors:** Fatemeh Mashhadiabbas, Sanaz Gholami Toghchi, Roohollah Safarpour

**Affiliations:** 1 Dept. of Oral and Maxillofacial Pathology, School of Dentistry, Shahid Beheshti University of Medical Sciences, Tehran, Iran; 2 Dept. of Oral and Maxillofacial Pathology, School of Dentistry, Lorestan University of Medical Sciences, Khorramabad, Iran

**Keywords:** Central granular cell odontogenic tumor, Central granular cell odontogenic fibroma, Odontogenic tumor, Granular cell ameloblastic fibroma

## Abstract

The central granular cell odontogenic tumor (CGCOT) is a rare, benign, slowly growing, odontogenic neoplasm. CGCOT was not considered as a distinct entity in the WHO classification reported on 2017. This study reports a rare case of CGCOT involving the right side of maxillary anterior region of a 39-year-old white woman. In addition, to better delineate the clinical, radiographic, histopathologic and immunohistochemical characteristics of CGCOT, a literature review of all published cases (in PubMed/ Google Scholar/ MEDLINE/Scopus) of CGCOT is provided. CGCOT is a very uncommon tumor, with only 51 reported cases in the literature. The present case is interesting regarding to its rarity for being in the maxillary anterior region, which has not been previously reported in Asia. The immunohistochemical findings of the current case and other cases in the literature review, verified the mesenchymal origin of granular cells and odontogenic nature of the epithelium islands, which can be a possible promise for placing this lesion in the future WHO odontogenic tumor classification.

## Introduction

The rare granular cell odontogenic tumor (GCOT) was primarily reported by Werthemann in 1950 [ 1 ], named as sponginocytic adamantinoma. There are immense controversies concerning the notion and the definition of this lesion. This lesion has been differently named as granular cell ameloblastic fibroma [ 2 ], ameloblastic fibroma with stroma of granular cells [ 3 ], central granular cell tumor of the jaw [ 4 ], central granular cell odontogenic fibroma [ 5 ], central odontogenic fibroma (granular cell variant) [ 6 ], central odontogenic granular cell tumor (COGCT) [ 7 ], central granular cell odontogenic tumor (CGCOT) [ 8 ], and finally GCOT [ 9 ].

Even though WHO proposed the term CGCOT for this lesion [ 10 ], there is still a great debate on this nomenclature since it was not considered as a distinct entity in the recent WHO classification [ 11 ] of odontogenic tumors. However, recent published studies suggest the term CGCOT for tumors characterized by varying amount of large eosinophilic granular cells with eccentrically placed nuclei associated with apparently inactive odontogenic epithelium [ 8 , 12 - 24 ]. CGCOT is defined as a rare, benign, slow-growing, noninvasive, though non-encapsulated odontogenic neoplasm [ 25 ]. This lesion is usually detected in the posterior mandible of women, predominantly in the fifth decade of life [ 20 ]. An extraosseous variant [ 26 , 27 ] and a malignant case of central granular cell odontogenic fibroma has also been reported [ 28 ].

Herein, we report the new rare case of CGCOT in the anterior area of maxilla in a 39-year-old female. Subsequently, we provide a literature review of all published cases (51 cases) of CGCOT.

## Case Presentation

**Figure 1:**
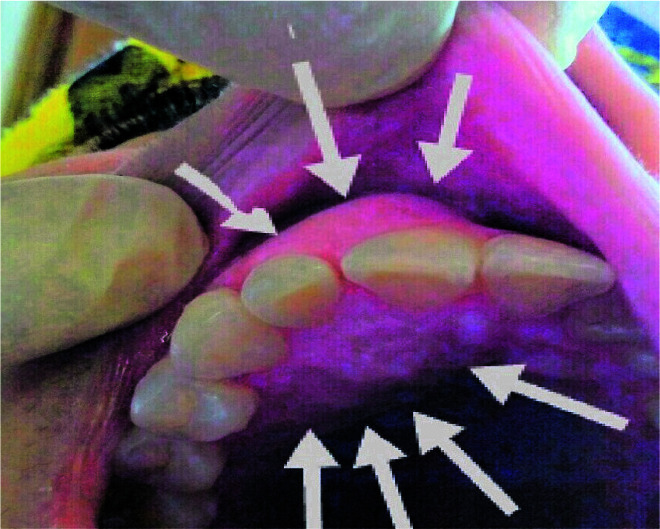
Clinical view showing a swelling on palatal and labial area of incisors/canine in maxilla (white arrows)

**Figure 2:**
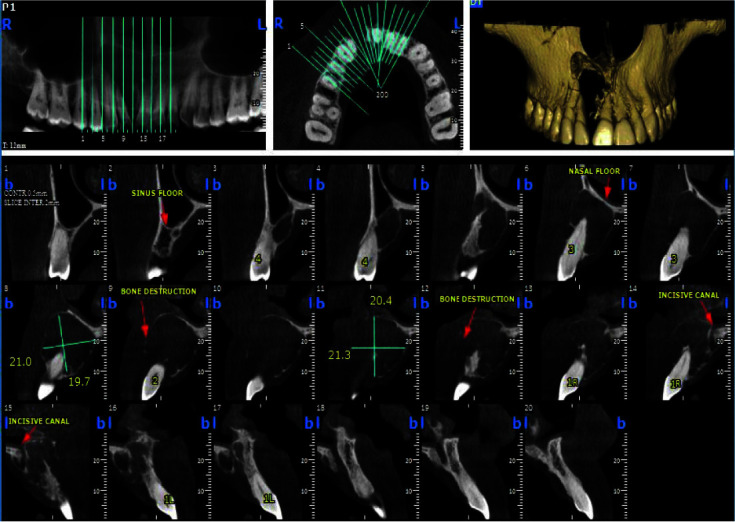
Cone beam computed tomography (CBCT) images show a well-defined corticated unilocular radiolucent lesion from maxillary right central incisor to the right first premolar

A 39-year-old white woman with a chief complaint of two- week history of painless swelling in the anterior region on right side of the maxilla was examined. A noticeable intra oral hard, asymptomatic swelling in the palatal and buccal area of maxilla extending from maxillary right central incisor to the first premolar was detected (Figure 1). The overlying mucosa of the region was smooth with normal color. The patient reported negative history of trauma, infection, prior tumors or any instance of radiation. All teeth in the quadrant showed a positive response to vitality test. The cone beam computed tomography (CBCT) sans showed a well-defined corticated unilocular radiolucent lesion measuring 21.3×20.4mm from maxillary right central incisor to the first premolar, causing expansion, thinning of palatal and labial cortex, and divergence between central and lateral incisor roots (Figure 2).

**Figure 3:**
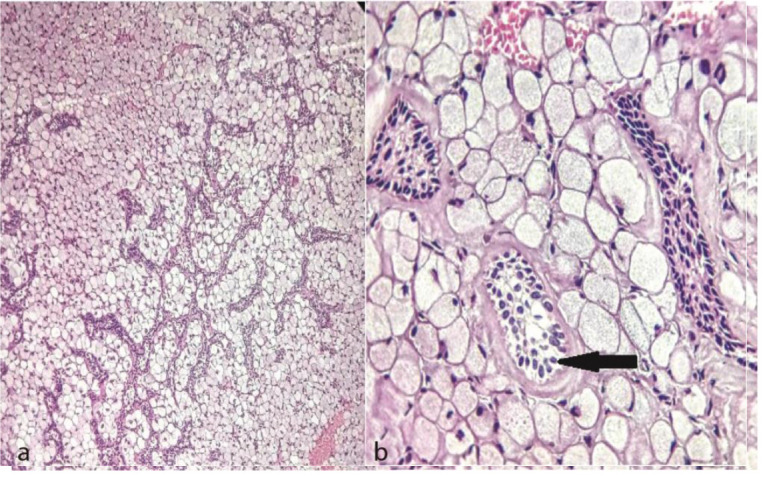
Histopathologic sections show, **a:** Sheets and lobules of eosinophilic granular cells intermixed with odontogenic epithelial cords and strands (H&E, original magnification 100×), **b:** Large granular cells with eccentric placed nuclei and odontogenic epithelium with vacuolated changes (black arrow) (H&E, original magnification 400×)

**Figure 4:**
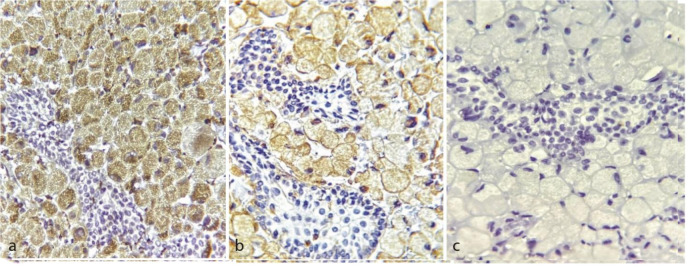
**a:** CD68 staining; granular cells show positive immunostaining, and the odontogenic epithelium is negative (original magnification 400×), **b:** Vimentin staining; granular cells show positive immunostaining, whereas the odontogenic epithelium shows no immunoreactivity (original magnification 400×), **c:** S-100 staining; granular cells are negative for S-100 protein (original magnification 400×)

The aspiration examination of the lesion was negative. Regarding the clinical, radiological, and aspiration examinations, odontogenic tumors including ameloblastoma and odontogenic myxoma were considered in our differential diagnosis list. Afterwards, an incisional biopsy was performed for histopathological examination. Grossly, the specimen was multiple pieces of irregular, gray-brown soft tissue, measuring 1.6×1.3×0.4cm. Incut surface, the lesion was creamy-gray, homogeneous and solid. Microscopic examination of Hematoxylin and Eosin (H&E) stained soft tissue sections discovered a benign mesenchymal odontogenic neoplasm with lobulated pattern containing large polygonal cells abundant pale eosinophilic, granular cytoplasm, and eccentric vesicular nuclei. Narrow cords and nests of odontogenic epithelium that were scattered among the granular cells were observed (Figure 3a-b). On immunohistochemical (IHC) staining, the granular cells showed positive expression for CD68 antigen (Figure 4a) and vimentin (Figure 4b) and negative expression for S-100 protein (Figure 4c). Regarding the histopathological and immunohistochemical findings, an accurate diagnosis of CGCOT was made. Informed consent was obtained from the patient for the information required to report the case. Unfortunately, because of financial limitations, the patient did not return for further treatment and therapeutic surgery. 

### Search strategy for literature review

As searching strategy, several databases (PubMed/ Google Scholar/ MEDLINE/Scopus) were searched for case reports and case series reported since September 2021, with using combinations of the keywords including central granular cell odontogenic tumor, granular cell ameloblastic fibroma, odontogenic tumor and central granular cell odontogenic fibroma. We screened the title and abstract for manuscript selection. Reference lists from the citations were also reviewed for the relevant publications. We found 36 reports [ 1 - 9 , 12 - 24 , 29 , 42 ] including 51 cases with certified histopathological diagnosis of CGCOT or suggestive histopathological features of CGCOT which has been reported with other terminologies for the present review. These studies are collected in Tables 1-2.

**Table 1:** Characteristics of reported cases of central granular cell odontogenic tumor (CGCOT), 1950-2021

	Author(s)	Year	Age yrs.	Gender	Location	Radiographic features	Treatment	Follow-up (m/yrs.)
1	Werthemann [ 1 ]	1950	39	M	Left mandibular premolar/molar	NS	NS	NS
2	Couch *et al*. [ 2 ]^A^	1962	55	F	Left mandibular/ second molar	Radiolucent lesion with loculated borders	Conservative removal of the lesion with tooth extraction	NR 8 m
3	Couch *et al*. [ 2 ]^B^	1962	59	F	Left mandibular/ canine	Loculated radiolucency with focal densities	Removal of tumor	NR 27 m
4	Waldron *et al*. [ 29 ] ^A^	1963	60	F	Left mandibular/ canine	2.0 cm radiolucent lesion	Removal of tumor	NR 29 m
5	Waldron *et al*. [ 29 ]^B^	1963	53	F	Left mandibular/ molar	2.0–3.0 cm cystic radiolucency displacing teeth	Removal of the mass with tooth extraction	NR 3 m
6	Gorlin and Goldman [ 30 ]	1970	50	F	Mandibular molar region	NS	Curettage	NS
7	Dalforno and Donna [ 3 ]	1970	57	NS	Left mandibular/ molar	NS	Curettage	NR 6 m
8	White *et al*. [ 4 ] ^A^	1978	50	F	Mandibular canine area	Radiolucency	Curettage	NR 6 m
9	White *et al*.[ 4 ] ^B^	1978	50	F	Mandibular posterior area	Radiolucency	Curettage	NR 7 yrs.
10	White *et al*.[ 4 ] ^C^	1978	55	F	Maxillary premolar	Radiolucency	Surgical excision	NR 3 yrs.
11	White *et al*.[ 4 ] ^D^	1978	65	F	Mandibular premolar/molar	Radiolucency	Surgical excision	NR 2 yrs.
12	Regezi *et al*. [ 31 ]^A^	1978	29	F	Maxilla	NS	NS	NS
13	Regezi *et al*. [ 31 ]^B^	1978	16	M	Mandible	NS	NS	NS
14	Vincent *et al*. [ 4 ]^A^	1987	51	F	Right mandibular premolar/ molar	4–2 cm radiolucency with sclerotic border	Conservative removal of the mass	NS
15	Vincent *et al*.[ 5 ] ^B^	1987	27	M	Right mandibular second premolar/first molar	1.5 cm unicystic radiolucency with sclerotic borders	Surgical excision	NR 24 m
16	Shiro *et al*. [ 6 ]	1989	45	F	Left mandibular premolars	0.7–0.4 cm unicystic radiolucency	Surgical excision	NR 4 yrs.
17	Mirchandani *et al*. [ 7 ]	1989	33	F	Mandible	radiolucency	NS	NS
18	Ruhl and Akuamoa-Boateng [ 32 ]	1989	22	M	Left maxillary first and second molars	4.5 cm with slight displacement of teeth	En bloc resection	NS
19	Chen [ 33 ]^A^	1991	50	F	Right mandibular canine	1.0–0.8 cm radiolucency	NS	NS
20	Chen [ 33 ]^B^	1991	45	F	Left mandibular premolar/molar	5.0–3.0 cm radiolucency	NS	NS
21	Chen [ 33 ]^C^	1991	64	F	Left mandibular canine/premolar	3.0–2.0 cm radiolucency	NS	NS
22	Chen [ 33 ]^D^	1991	77	F	Left mandibular/ premolars	0.5–0.5 cm radiolucency	NS	NS
23	Yih *et al*. [ 38 ]	1995	66	F	Left mandibular/ second premolar	0.5–0.5 cm unilocular radiolucency	Curettage	NR 6 m
24	Gesek *et al*. [ 8 ]	1995	62	F	Left mandibular/ second premolar	Multilocular, well circumscribed radiolucency	Curettage	NR 12 m
25	Machado de Sousa *et al*. [ 39 ]^A^	1998	19	F	Right maxillary premolar/molar	Well-delineated multilocular radiolucency	Surgical excision	NR 24 m
26	Machado de Sousa *et al*. [ 39 ]^B^	1998	25	M	Right maxillary premolar/molar	8.0 cm radiopaque lesion	Surgical excision	NR 120 m
27	Ardekian *et al*. [ 12 ]	1998	63	M	Right maxillary premolar/molar	Well-defined radiolucency with sclerotic border	Curettage Teeth extraction	NR 48 m
28	Matsumoto *et al*. [ 34 ]	2000	24	M	Left mandibular/ premolars	Well demarcated radiolucent lesion	Enucleation with teeth extraction	NR 1.5 yrs.
29	Brannon *et al*.[ 13 ]^A^	2002	36	F	Mandibular canine/ premolar	NS	NS	NS
30	Brannon *et al*.[ 13 ]^B^	2002	50	F	Jaw, NS	NS	NS	NS
31	Brannon *et al*.[ 13 ]^C^	2002	32	F	Mandibular canine/premolar	Multilocular radolucency with sclerotic border	Teeth extracted with surgical excision	NR 180 m
32	Brannon *et al*.[ 13 ]^D^	2002	19	F	Left maxillary first premolar /first molar	Unicystic radiolucency enveloping roots of second premolar	Curettage	R 156 m
33	Brannon *et al*.[ 13 ]^E^	2002	48	M	Right side of maxilla	NS	NS	NS
34	Calvo *et al*. [ 40 ]	2002	61	M	Anterior region of maxilla	Radiolucency with resorption of anterior teeth	NS	NS
35	Meer *et al*. [ 14 ]	2004	65	F	Left mandibular first premolar/ second molar	Irregular radiolucency from first premolar to second molar	Surgical excision	NR 12 m
36	Reichart *et al*. [ 41 ]	2006	46	F	Right mandibular premolar/molar	Multilocular radiolucent lesion	Surgical excision with reconstruction	NR 2 yrs.
37	Gomes *et al*. [ 9 ]	2006	20	F	Left mandibular premolars/ molars	An intra-osseous mixed lesion,5 cm	Enucleation	NR 7 m
38	Kim *et al*. [ 15 ]	2006	33	M	Right maxillary premolar /first molar	Well-defined unilocular radiolucency	Enucleation with tooth extraction	NR 23 m
39	Mesquita *et al*. [ 16 ]	2009	20	F	Left mandibular second premolar/second molar	Well-defined radiolucency with foci of calcifications	Complete resection of the tumor	NR 4 yrs.
40	Lotay *et al*. [ 42 ]	2010	28	F	Right maxillary/ premolar	1.5–2.5 cm well-defined mixed lesion	Enucleation and curettage	NS
41	Silva *et al*. [ 17 ]	2012	41	F	Left side of maxilla	Well-defined mixed lesion	Surgical excision	NR 2 yrs.
42	Sarode *et al*. [ 18 ]	2013	25	F	Right side of mandible crossing the midline	Well-demarcated multilocular radiolucent lesion	Enucleation and curettage	NR 2 yrs.
43	Cheng *et al*. [ 19 ]	2013	52	F	Right mandibular/ premolars	Well-defined mixed lesion	Enucleation	NR 3 m
44	Chiang *et al*. [ 20 ]	2014	69	M	Left side of the mandible, ramus	well-demarcated radiolucent lesion	Surgical excision	NR 2 m
45	Anbiaee *et al*. [ 21 ]	2014	16	F	Left mandibular angle	Multilocular mixed lesion,3×5cm	Surgical resection with mandibular reconstruction	NR 2 yrs.
46	Lee *et al*. [ 22 ]	2014	19	M	Left mandibular third molar	Enlarged dental follicle	Enucleation with the tooth extraction	NS
47	Fletcher *et al*. [ 35 ]	2015	19	F	Right mandibular second premolar/ molars	Unilocular radiolucent lesion	Curettage	NR 24 m
48	Vennamaneni *et al*. [ 36 ]	2016	38	M	Right mandibular premolars/first molar	Well defined unilocular radiolucent lesion	Enucleation	NR NS
49	Madan *et al*. [ 23 ]	2016	73	M	Anterior area of mandible	Multilocular radiolucent lesion	Segmental resection	NR 9m
50	Atarbashi *et al*. [ 37 ]	2019	57	F	Left mandibular premolars/ first molar	well- defined radiolucent lesion	Enucleation	NR 12m
51	Koth *et al*. [ 24 ]	2021	42	F	Left maxillary anterior	Unilocular radiolucency	Surgically removal	NR 16m

KEY: GCAF: granular cell ameloblastic fibroma; CGCOF: central granular cell odontogenic fibroma, CGCT: central granular cell tumor; COGCT: central odontogenic granular cell tumor; CGCOT: central granular cell odontogenic tumor; GCOT: granular cell odontogenic tumor; F: female; M: male; NS: not stated; NR: no recurrence; R: recurrence; M: months; yrs.: Years.

**Table 2:** Summary of clinical, pathological, and paraclinical results of reported cases

Total case Number	51
Year of publication	1950-2021
Age (years)	Mean, 43.53 y (range 16-77y)
Gender	Female, 36; Male, 14; Not stated, 1
Race	White, 16; Black, 10; Yellow, 1;Indian, 1; Oriental, 1; Afro-Caribbean, 1; Caucasian, 1; Not stated, 20
Site of lesion	Mandible, 37(premolar/molar area: 28, canine/ anterior region: 6, NS: 3); Maxilla, 13(premolar/molar area: 8, canine/ anterior region: 2, NS: 3); Not stated, 1
Signs and symptoms	Painless swelling, 24; Asymptomatic, 8; Painful and no swelling, 3; Not stated, 16
Radiographic features	Radiolucency, 34(unilocular: 28, multilocular: 6); Mixed lesion, 8; Opaque, 1; Not stated, 8
IHC markers	Positive GC; Mostly: Vimentin, CD 68 (Lesser: Lysozyme, AACT, AAT, B-cl2, CEA, NSE)
	Negative GC; S-100
	Positive OE; Mostly: CK 14 (Lesser: CK 13, Pan CK, B-cl2, CK 5, CK 7, CK 8)
Treatment	Enucleation and/or Curettage, 24; Surgical resection, 15; Not stated, 12
Follow-up	Mean, 33 m (range 2-180 m) ; Not stated 17

KEY: m: months; y: years; IHC markers: Immunohistochemical markers; GC: granular cell; OE: odontogenic epithelium; AACT: α1-antichymotrypsin; AAT: α1-antitrypsin; NSE, *CEA: carcinoembryonic antigen*; *NSE:* neuron specific enolase; CK: cytokeratin

## Discussion

CGCOT is considered as an imperative, yet rare, odontogenic tumor. In 1950, Werthemann [ 1 ] first described this lesion in the left side of the mandible and defined it as spongiocytic adamantinoma. Histopathologically, he described this lesion as comparatively large, bright, rounded, and rather polyhedral cells with small nuclei, which were mostly located on the periphery of the cell body, intermixed with epithelial cones and cords. In 1962, Couch *et al*. [ 2 ] described two cases of central jaw lesions, which were composed of granular cells allied with nests of odontogenic epithelium on microscope. They and some other investigators named this lesion as granular cell ameloblastic fibroma [ 2 , 29 - 32 ].

Dalforno and Donna [ 3 ] defined this lesion as ameloblastic fibroma with stroma of granular cells. Later, other investigators named this tumor as central granular cell tumor of the jaws [ 4 , 33 ], central granular cell odontogenic fibroma [ 5 , 7 ], central odontogenic fibroma, granular cell variant [ 6 , 40 - 41 ] and COGCT [ 34 , 38 - 39 ]. At present, most researchers rather to name this lesion as CGCOT [ 8 , 12 - 24 ]; we also prefer this term. Moreover, four authors describe this lesion as GCOT [ 9 , 35 - 37 ].

The review of literature showed that the mean age of the 51 reported cases was 43.53 years with a range of 16 to 77 years. The mean age was reported in previous researches as 47.3 in Gesek *et al*. [ 8 ], 46. 2 in Gomes *et al*. [ 9 ], 45.8 in Chiang *et al*. [ 20 ], and 45.21 in Sarode *et al*. [ 10 ]; which were higher than the age of our case. Our review showed that more than half (61%) of patients were older than 40 years of age, which is similarly reported by Sarode *et al*. [ 10 ] and Neville *et al*. [ 43 ]. There is a marked female predilection (72%) in this lesion. The most common location was the mandibular premolar/ molar area (64%), followed by maxillary premolar / molar area (18%). Only two cases affecting the anterior region of maxilla (4.5%) was reported [ 24 , 40 ]. In mandible, there was a tendency for tumor growth on the left side (20 cases, 69%), in contrast to the right side (9 cases, 31%).In maxilla, this tendency occurred in the right side of the jaw . However, Chiang *et al*. [ 20 ] described equal distribution of this tumor on the left side and right side of the maxilla. Our review showed that 52% of cases affected whites, which was similarly reported by Kim *et al*. [ 15 ] and Chiang *et al*. [ 20 ].

Clinically, most lesions (24 cases, 68.5%) presented as a asymptomatic mass with localized expansion, and some lesions were completely asymptomatic (8 cases, 23%).Only three cases (8.5%) presented as a painful lesion without swelling [ 17 , 37 , 39 ].

The current case is the first reported case of CGCOT in Asia that occurred in the maxillary anterior region, a very rare location, while other features of our case were approximately similar to most previous studies.

The literature review revealed that the most common radiological finding was a unilocular radiolucent lesion (28 cases, 65%), similar to our case. Some lesions presented as a mixed radiolucent-radiopaque lesion (8 cases, 18.5%), or multilocular radiolucency (6 cases, 14%). Only one case (2.5%) presented as a radiopaque lesion in appearance [ 41 ]. Extraosseous variant of GCOT is rarer than its central type. To our knowledge, only four cases of GCOT have been described in the gingival soft tissues [ 26 - 27 , 44 - 45 ].

Histopathologically, this odontogenic tumor is characterized by varying amount of large eosinophilic granular cells with eccentrically placed nuclei associated with apparently inactive odontogenic epithelium [ 1 - 9 , 12 - 24 , 29 - 42 ], which was also found in our case. Epithelial cells containing a clear cytoplasm were a common feature in the reported studies [ 6 , 13 , 16 - 20 , 41 - 42 ]. Cementum-like material [ 2 , 4 , 8 , 13 , 29 , 31 , 39 ], dystrophic calcifications [ 33 ] and palisading or polarization of the peripheral epithelial cells were also reported [ 8 ].

On IHC examinations, granular cells showed positive immunoreactivity for vimentin (29%) and CD 68 (29%) and negativity for cytokeratin (CK) in all the collected cases. These findings suggest mesenchymal origin of GCs. On the other hand, immunoreactivity for S-100 protein in granular cells was reported negative in all cases, which suggests a non-neural, mesenchymal origin for this tumor. Odontogenic epithelium shows variable expression of CK. Our review showed that CK 14(19%) had the most positive immunoreactivity, followed by CK 13, Pan CK, b-cl2 and AE1. The histopathological differential diagnosis can be considered as GCT of soft tissue, granular cell variant of ameloblastoma and congenital epulis [ 7 ]. GCT does not show odontogenic islands, cementum-like material, or dystrophic calcification and is strongly positive for S-100 protein [ 32 ]. Granular cells in granular cell ameloblastoma are immuno-positive for CK, but negative for S-100 protein. The histological and immunohistochemical aspects of congenital epulis of newborns are comparable to CGCOT, but dissimilar ages of patients who were involved with congenital epulis as well as the location of this lesion (alveolar ridge) are expedient for final diagnosis [ 32 ].

Our review revealed that 24 cases (61.5%) received excision and/or curettage for their treatment, while surgical removal with reconstruction of jaw was performed in 15 cases (38.5 %). The prognosis of this tumor is good; 33 cases (97%) reported no evidence of recurrence, with the range of follow up time from 2 to 180 months). Only one case recurred 13 years after initial treatment [ 13 ]. Piattelli *et al*. [ 28 ] in 2003 reported the first and the only case of malignancy in this tumor. However, no case of metastasis has been reported until now. 

## Conclusion

CGCOT is a rare tumor with only 51 reported cases in the literature. The presented case is rare concerning its location on maxillary anterior region, which has not been yet reported in Asia. IHC findings of the current case and other cases in the present review, confirmed the mesenchymal origin of GCs and odontogenic nature of the epithelium islands, a prominence that necessitates its assignment in the future WHO odontogenic tumor classification.

## Conflict of Interest

The authors declare that they have no conflict of interest.
